# Povidone-iodine-induced transient triiodothyronine thyrotoxicosis in a Japanese patient with prolonged habitual gargling: A case report and literature review

**DOI:** 10.1097/MD.0000000000034631

**Published:** 2023-08-25

**Authors:** Ryuji Suzuki, Seiji Suzuki

**Affiliations:** a Department of Internal Medicine, Hitotsubashi Hospital, Tokyo, Japan; b Emeritus Professor at Showa University, Tokyo, Japan.

**Keywords:** iodine-induced hyperthyroidism, non-thyroid disease, povidone-iodine, triiodothyronine thyrotoxicosis

## Abstract

**Rationale:**

Iodine-induced hyperthyroidism and triiodothyronine (T3) thyrotoxicosis in patients who routinely gargle with povidone-iodine (PVP-I) gargling solution are rare in Japan.

**Patient concerns:**

A 50-year-old man presented to our hospital for a close examination of an enlarged thyroid, which was noted during a complete health checkup. The thyroid was slightly enlarged with no palpable nodules. He had an increased appetite but no weight gain. He had been routinely gargling with PVP-I gargling solution 4 times daily for >10 years. He had no history of thyroid disease.

**Diagnoses:**

Test results revealed suppressed thyroid-stimulating hormone, normal free thyroxine, and increased free triiodothyronine levels, leading to the diagnosis of T3 thyrotoxicosis.

**Interventions:**

The patient agreed to stop gargling with PVP-I gargle solution.

**Outcomes:**

The free triiodothyronine and thyroid-stimulating hormone levels returned to normal at 18 and 21 weeks, respectively, after discontinuation of PVP-I gargling. After an improvement in thyroid function, he gained 5 kg in 1 year.

**Lessons:**

To our knowledge, this is the first case report that describes PVP-I gargle-induced T3 thyrotoxicosis in a healthy individual without thyroid disease. In Japan, which is an iodine-sufficient country, considering the possibility of high-dose iodine intake-induced thyrotoxicosis due to long-term PVP-I gargling or other causes is necessary, even in individuals with no history of thyroid disease.

## 1. Introduction

Povidone-iodine (PVP-I) gargles have been commonly used in Japan and other countries for decades.^[1]^ PVP-I gargles marketed in Japan contain 7 mg of effective iodine in 1 mL of undiluted solution.^[1]^ Gargling with 4 mL of the solution containing 7% PVP-I results in the absorption of approximately 4.2 mg or 10% of total iodine through the oral mucosa or gastrointestinal tract.^[2]^ While Graves’ disease, multinodular disease, iodine-deficiency goiter, and Hashimoto’s thyroiditis have been described as predisposing factors for hyperthyroidism caused by iodine overdose, limited reports exist on iodine-induced hyperthyroidism in the absence of thyroid disease.^[3,4]^ Reports of triiodothyronine (T3) thyrotoxicosis are even rarer.

Here, we report a Japanese man with transient T3 thyrotoxicosis developed due to habitual gargling with a 7% PVP-I gargling solution for >10 years. The patient had no underlying thyroid disease.

## 2. Case presentation

A 50-year-old man presented to our hospital for a close examination of an enlarged thyroid gland, which was noted during a complete health checkup. He had an increased appetite but no weight gain. His social history was negative for alcohol consumption or smoking. His medical history was significant for hyperlipidemia, and family history, for hypertension and diabetes mellitus. The patient had no history of thyroid disease. The patient was taking antihyperlipidemic medication (statin). He had routinely gargled with 7% PVP-I gargling solution (30 mL/bottle, Isodin Gargle) 4 times daily for >10 years. Informed consent was obtained from the patient for the publication of this case.

On examination, he was 63.9 kg and 176.1 cm tall, with a body mass index of 20.6 kg/m^2^. His blood pressure was 113/68 mm Hg, with a pulse rate of 84 beats/min. The thyroid was slightly enlarged with no palpable nodules. He had no history of ophthalmopathy. The thyroid-stimulating hormone (TSH) level was suppressed to 0.01 μIU/mL (reference range 0.46–3.50 μIU/mL), and the free triiodothyronine (FT3) level had increased to 5.1 pg/mL (reference range 2.2–4.1 pg/mL). His free thyroxine (FT4) level was 1.1 ng/dL (reference range 0.8–1.6 ng/dL). The hormonal profile indicated T3 thyrotoxicosis. The patient tested negative for antithyroid-stimulating hormone receptor (third generation), antithyroid peroxidase (anti-TPO), and antithyroglobulin (anti-TG) antibodies. Thyroid ultrasonography revealed enlarged and poorly perfused lobes. Internal echogenicity was somewhat uneven, and small cysts were observed in the lobes. He agreed to stop gargling with the 7% PVP-I gargling solution. Therefore, a non-iodine-containing gargling solution (azulene sulfonate-containing solution: Azunol) was prescribed.

Blood tests performed 91 days after PVP-I gargling discontinuation still indicated T3 thyrotoxicosis persistence with TSH, 0.01 μIU/mL; FT3, 4.9 pg/mL; FT4, 1.1 ng/dL; antithyroid-stimulating hormone receptor antibody (third generation) < 0.8 (reference range < 2.0); thyroglobulin, 32.1 ng/mL (reference range ≦ 32.70 ng/mL); anti-TPO antibody < 9.0 (reference range < 16.0); and anti-TG antibody, 10.1 (reference range < 28.0). One hundred days after the PVP-I gargling discontinuation, technetium-99^m^ thyroid scintigraphy was performed at another hospital, which showed thyroid uptake of 0.3% (normal range 0.5%–4%), indicating thyroid suppression. This finding and poor perfusion in the lobes on thyroid ultrasonography ruled out Graves’ disease.

**Figure 1.**
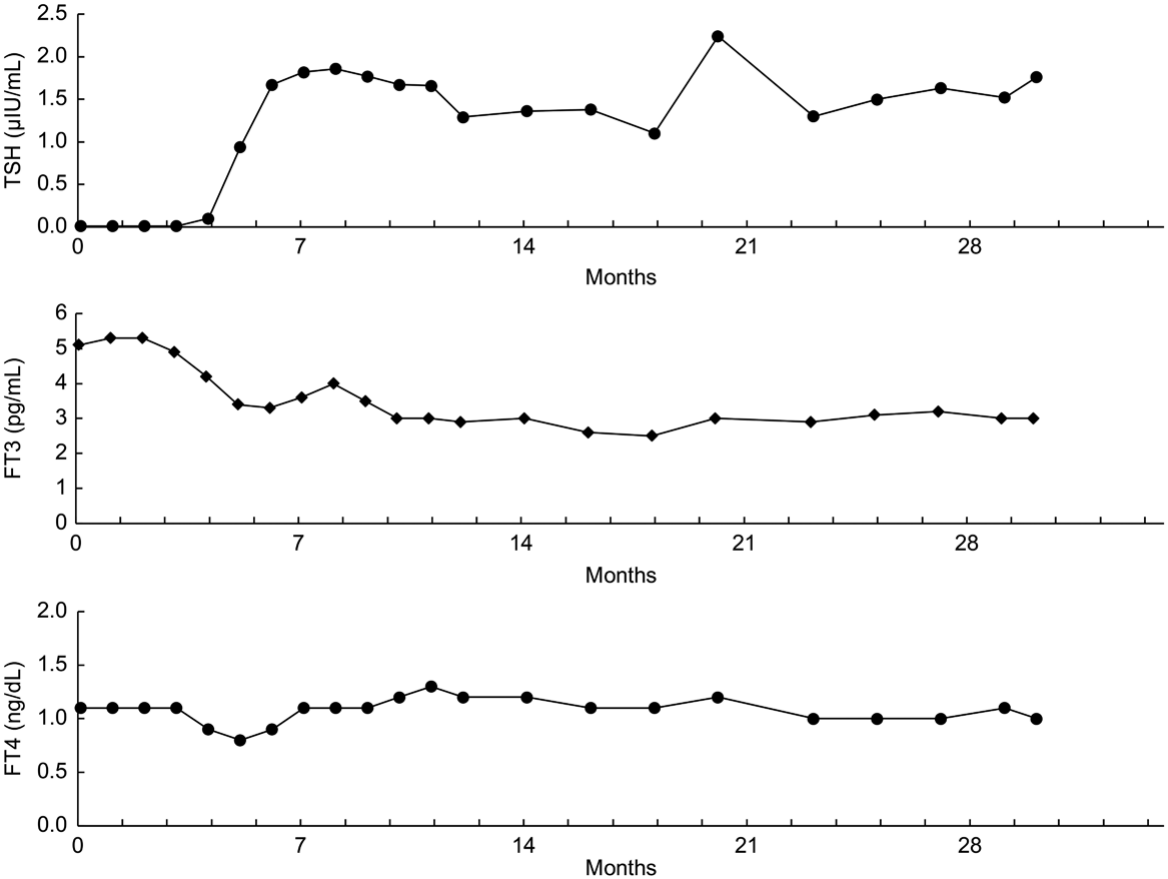
Patient’s clinical course. FT3 = free triiodothyronine, FT4 = free thyroxine, TSH = thyroid-stimulating hormone.

Blood tests performed 18 weeks (126 days) after the discontinuation showed FT3 levels within the normal range (TSH, 0.17 μIU/mL; FT3, 3.9 pg/mL; and FT4, 0.9 ng/dL). Thyroid radioiodine scintigraphy performed 128 days after the PVP-I gargling discontinuation at another hospital showed a 5% decrease in radioiodine uptake at 24 hours. Blood tests performed 21 weeks (147 days) after the PVP-I gargling discontinuation showed TSH levels within the normal range (TSH, 0.94 μIU/mL; FT3, 3.4 pg/mL; FT4, 0.8 ng/dL; thyroglobulin, 28.6 ng/mL), indicating improvement in T3 thyrotoxicosis. Thyroid ultrasonography performed 21 weeks (147 days) after the PVP-I gargling discontinuation showed a reduction in the short axis of the lobes and a normalization of blood flow in the lobes compared to the corresponding findings immediately after gargling discontinuation. The improvement in thyroid volume was attributed to the discontinuation of high-dose iodine loading. Thereafter, the patient’s thyroid autoantibody titers have been negative throughout the disease course, and he has been euthyroid for >2 years (Fig. 1). After an improvement in thyroid function, he gained 5 kg in 1 year.

## 3. Discussion

Our patient showed decreased radioiodine uptake and no increase in blood flow on color Doppler thyroid ultrasonography, suggesting the need to differentiate between painless thyroiditis, a common condition, and iodine-induced hyperthyroidism. Painless thyroiditis is generally associated with a family history of autoimmune thyroid disease and is characterized by positive thyroid autoantibodies such as anti-TPO and anti-TG antibodies.^[5]^ In the present case, the patient tested negative for thyroid autoantibodies throughout the disease course. Moreover, painless thyroiditis occurs when the thyroid tissue is destroyed, and thyroid hormones leak into the blood, and it is usually associated with increased blood levels of both T3 and thyroxine, but not T3 alone. The patient had T3 thyrotoxicosis, with FT4 levels within the normal range throughout the disease course (Fig. 1), and the FT3/FT4 was >3.1^[5]^; these findings are inconsistent with painless thyroiditis.

Iodine-induced hyperthyroidism is observed in iodine-deficient areas and is uncommon in Japan, an iodine-sufficient country where excessive iodine intake appears to be common.^[6]^ In Japan, iodine-induced hyperthyroidism is rare in patients without thyroid disease,^[6]^ but thyrotoxicosis induced by the excessive consumption of Japanese kombu (kelp) has been reported.^[7]^ T3 thyrotoxicosis is extremely rare.^[8]^ In healthy individuals without thyroid disease, short-term gargling with PVP-I is generally safe, but long-term gargling with PVP-I has been reported to induce thyroid dysfunction.^[1,2]^ Reported cases of PVP-I gargle-induced thyroid dysfunction in healthy individuals include hypothyroidism^[1,9]^; reports of iodine-induced hyperthyroidism are rare.

We reviewed the findings of thyroid scintigraphy and thyroid color Doppler ultrasonography in previously reported cases of iodine-induced hyperthyroidism in patients without a history of thyroid disease (Table 1).^[3,7,10–18]^ Although many reports describe decreased radioiodine intake,^[10–12,16]^ radioiodine uptake varies widely in cases of amiodarone-induced hyperthyroidism type 1.^[6]^ In iodine-induced hyperthyroidism, color Doppler thyroid ultrasonography generally shows increased blood flow^[6,14]^; however, cases without increased blood flow also exist.^[3,10,18]^

**Table 1:** Cases of iodine-induced hyperthyroidism with no history of thyroid diseases.

No.	Reference no.	Age (yr)	Sex	Iodine source	Time from discontinuation of iodine loading to improvement of thyroid function	Blood flow on thyroid ultrasonography	Thyroid scintigraphy	Increased FT3/FT4
1.	10	51	Female	Disinfection with PVP-I-impregnated cotton balls after urinary diversion with a Koch pouch	8 wk	No increase	Decreased (iodine)	Both
2	11	36	Male	Iodine-containing seaweed	9 wk	Not reported	Decreased (iodine)	Both
3	11	52	Male	Iodine-containing seaweed	11 wk	Not reported	Decreased (iodine)	Both
4	12	72	Female	Kelp-containing drug	6 mo	Not reported	Decreased (iodine) Normal (technetium)	Both
5	13	27	Female	Kelp-containing supplement	7 wk	Not reported	Not reported	Only T3 increased
6	14	45	Female	Kelp-containing drug	2 mo	Increased	Not reported	Both
7	15	70	Female	Kelp-containing supplement	Administered methimazole and improvement	Not reported	Not reported	Both
8	3	60	Female	Iodine-containing supplement	Administered methimazole and improvement	Normal	Iodine uptake on scintigraphy decreased 4 mo after supplement discontinuation	Both
9	7	42	Female	Japanese kombu	1 mo	Not reported	Not reported	Both
10	7	59	Female	Japanese kombu	1 mo	Not reported	Not reported	Both
11	16	30	Male	Dressing of the pressure sores with PVP-I	Administered neomercazol and improvement	Not reported	Decreased (iodine)	T3 at the upper limit of normal range, T4 increased
12	17	53	Female	CT scan with intravenous contrast	Developed 2 mo after contrast administration and improved in 2 wk	Not reported	Not reported	Both
13	18	17	Male	CT scan with intravenous contrast	Developed 5 d after contrast administration and improved with methimazole and steroids	No increase	Not reported	Both
14	The present case	50	Male	PVP-I gargle solution	18 wk	Decreased	Decreased (iodine) Decreased (technetium)	Only FT3 increased

CT = computed tomography, FT3 = free triiodothyronine, PVP-I = povidone-iodine, T3 = triiodothyronine, T4 = thyroxine.

To the best of our knowledge, only 2 cases of T3 thyrotoxicosis due to iodine-induced hyperthyroidism have been reported to date. Eliason reported a 27-year-old woman with T3 thyrotoxicosis who consumed an iodine-rich kelp-containing supplement, resulting in suppressed TSH and increased T3 levels with normal thyroxine levels.^[13]^ Moreover, Lee et al^[19]^ reported T3 thyrotoxicosis in 1 of 49 patients without thyroid dysfunction at baseline who received a single dose of contrast agent for computed tomography. This case is not included in Table 1 because their study was not a case report. They did not provide information on thyroid disease, such as the presence or absence of antithyroid antibodies.

## 4. Conclusion

To the best of our knowledge, this is the third case of iodine-induced T3 thyrotoxicosis and the first case of PVP-I gargle-induced T3 thyrotoxicosis in a healthy individual without thyroid disease, evaluated using thyroid scintigraphy and thyroid ultrasonography and followed up for 2 years. Even in Japan, an iodine-sufficient country, considering the possibility of high-dose iodine intake-induced thyrotoxicosis due to long-term PVP-I gargling or other causes is necessary, even in individuals with no family history of autoimmune thyroid disease or negative thyroid autoantibody test results.

## Acknowledgments

We are very grateful to the patient for participating in this study. We would like to thank Honyaku Center Inc. for English language editing.

## Author contributions

**Conceptualization:** Ryuji Suzuki, Seiji Suzuki.

**Data curation:** Ryuji Suzuki.

**Investigation:** Ryuji Suzuki, Seiji Suzuki.

**Supervision:** Ryuji Suzuki.

**Writing – original draft:** Ryuji Suzuki.

**Writing – review & editing:** Seiji Suzuki.
